# Petechial lesions in a patient with COVID-19^☆^^☆☆^

**DOI:** 10.1016/j.abd.2020.08.007

**Published:** 2020-11-26

**Authors:** Luciana Botinelly Mendonça Fujimoto, Silvana de Albuquerque Damasceno Ferreira, Fabiane Braga dos Santos, Carolina Talhari

**Affiliations:** a Department of Pathology and Legal Medicine, Universidade Federal do Amazonas, Manaus, AM, Brazil; b Department of Dermatopathology, Fundação Alfredo da Matta de Dermatologia e Venereologia, Manaus, AM, Brazil; c Private Clinic, Manaus, AM, Brazil; d Department of Dermatology, Universidade do Estado do Amazonas, Manaus, AM, Brazil

*Dear Editor,*

Rash, urticaria, and varicelliform presentations have already been associated with SARS-CoV-2.^1^, ^2^ Pernio-like lesions, classically associated with lupus erythematosus, have also been reported.^3^, ^4^

A 39-year-old female patient presented fever, dry cough, and odynophagia two weeks prior. After a presumptive diagnosis of COVID-19, she was treated with azithromycin, acetylcysteine, vitamin C, and zinc. On the tenth day, she presented anosmia, worsening of the cough, and painless, non-pruritic lesions on the fingers.

The patient reported hepatic steatosis and systemic arterial hypertension (she used losartan, atenolol, hydrochlorothiazide, and amlodipine, daily).

The physical examination revealed erythematous macules on the third, fourth, and fifth left fingers (Figure 1, Figure 2). Skin biopsy of the lesion of the fourth finger and swab (the same as that used to collect oro- and nasopharynx fluids) of the collected skin tissue were performed. After this procedure, the swab was placed in a tube with saline for reverse transcription polymerase chain reaction (RT-PCR).

**Figure 1:**
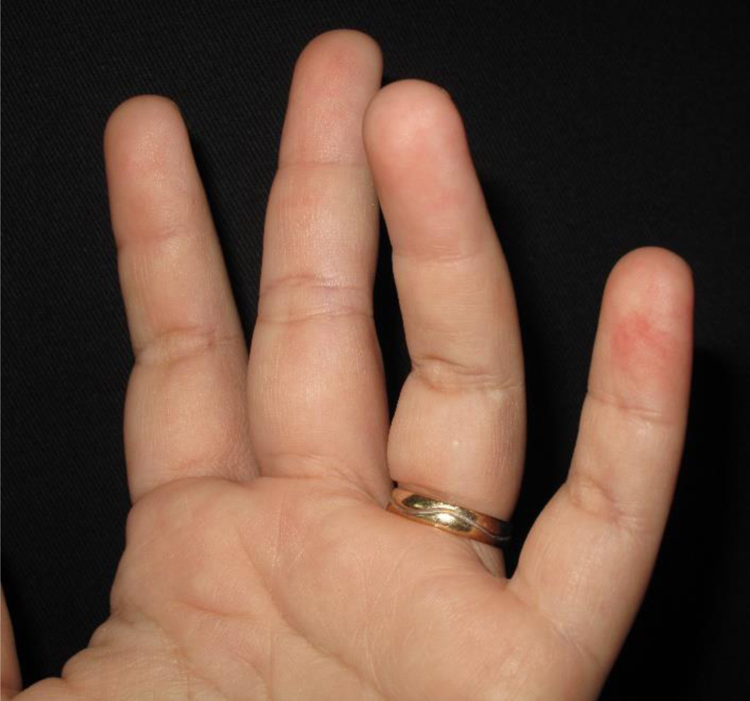
Erythematous macules on the digital pulps of the third, fourth, and fifth fingers of the left hand.

**Figure 2:**
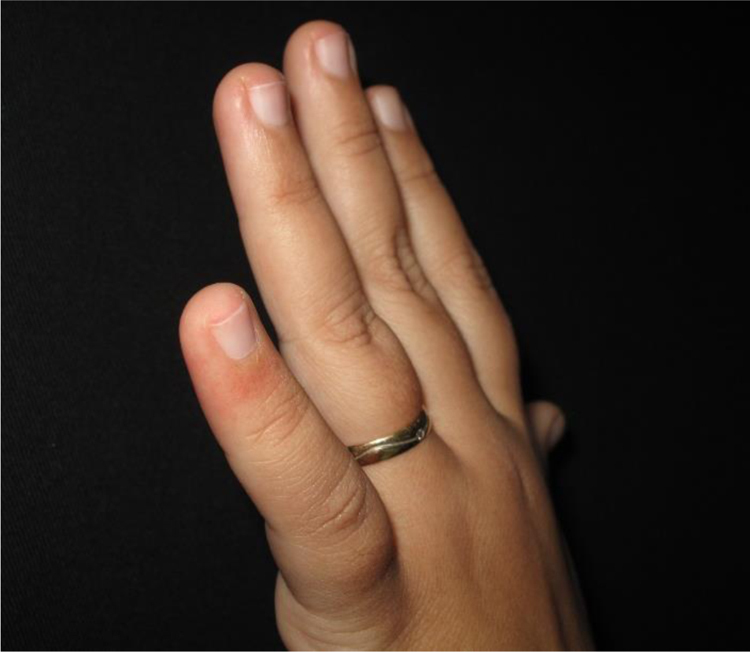
Erythematous macula on the dorsal surface of the fifth finger of the left hand.

Histopathological examination revealed a discrete focus of spongiosis in the epidermis, with slightly increased volume of keratinocytes, cytoplasmic vacuolization, and elongated and hyperchromatic nuclei. Apparently, there was pale intranuclear inclusion. In the dermis adjacent to these focal areas, there was a slight interface change with lymphohistiocytic permeation of the basal layer, perivascular inflammatory infiltrate, and vessels with intermingled nuclear debris (Fig. 3).

**Figure 3:**
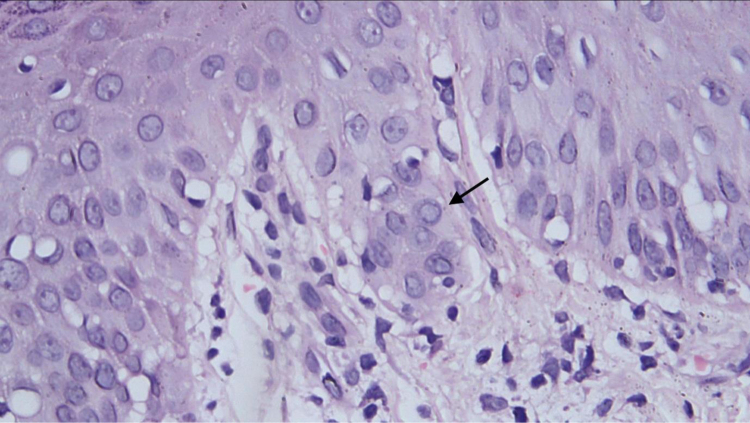
Pale nuclear inclusion (arrow) in epidermal cell amid spongiosis. Adjacent interface change with lymphocyte permeation and slight extravasation of red blood cells in the dermis (Hematoxylin & eosin, ×40).

The SARS-CoV-2 virus was not identified by RT-PCR in the samples collected from the nasopharynx and skin. The anti-SARS-CoV-2 IgM antibody in peripheral blood was reactive and the IgG was non-reactive. Serological tests for dengue fever (DENV), Zika (ZIKV), and chikungunya (CHKV) virus were negative.

The patient was treated with topical corticosteroids, three times a day, for five days, with disappearance of the cutaneous lesions.

In Italy, the majority of SARS-CoV-2 patients developed pernio-like lesions on the tenth day of illness, similar to the present case.^3^ However, more localized acral edema, pain, and/or itching was observed in more than 70% of cases.^3^, ^4^ There are reports of pernio-like lesions associated with COVID-19, with vacuolar changes in the basal layer with apoptotic keratinocytes and lichenoid inflammatory infiltrate in the papillary and reticular dermis.^4^ There is no description of a viral cytopathic effect, as in the case reported here.

There is still no gold standard technique for the identification of SARS-CoV-2 in the skin. In the autopsy of patients with COVID-19, a swab was introduced directly into the lung tissue. In that study, a sample collected was positive, by RT-PCR, for SARS-CoV-2.^5^ From this observation, a similar methodology was adopted for the present case.

In patients with skin rash, from tropical countries, several viruses should be investigated; DENV, ZIKV, and CHIKV are among the main ones. In the present case, there was also the possibility of a drug eruption, since in addition to antihypertensive drugs, the patient also used azithromycin and acetylcysteine before the onset of the skin condition. This hypothesis was ruled out by the histopathological examination. The IgM antibody reagent for SARS-CoV-2, negative serologies for DENV, ZIKV, CHIKV, and histopathological findings suggest that the lesions presented here are associated with COVID-19.

Patients with a clinical and/or laboratory picture of COVID-19 with cutaneous manifestations should be clinically and histopathologically evaluated by dermatologists, for the correct diagnosis and therapeutic conduct.

## Ethical aspects

This case report was submitted and approved by the Research Ethics Committee of Fundação Alfredo da Matta de Dermatologia (CAAE: 32573520.7.0000.0002). The patient signed an informed consent.

## Financial support

None declared.

## Authors’ contributions

Luciana Botinelly Mendonça Fujimoto: Approval of the final version of the manuscript; design and planning of the study; analysis and interpretation of data; critical review of the manuscript.

Silvana de Albuquerque Damasceno Ferreira: Approval of the final version of the manuscript; analysis and interpretation of data; critical review of the manuscript.

Fabiane Braga dos Santos: Approval of the final version of the manuscript; design and planning of the study; analysis and interpretation of data; critical review of the manuscript.

Carolina Talhari: Approval of the final version of the manuscript; design and planning of the study; analysis and interpretation of data; editing of the manuscript; critical review of the manuscript.

## Conflicts of interest

None declared.
